# Psychological status of healthcare workers in the post-COVID 19 period in China: a retrospective multicentric cross-sectional study

**DOI:** 10.3389/fpsyt.2025.1520361

**Published:** 2025-04-07

**Authors:** Xiaoli Wang, Fang Fu, Aihua Gan, Yuqian Wu, Wenjing Pan, Xin Zhou, Xiaohui Zhang

**Affiliations:** ^1^ Division of Academic Affairs, Huizhou Health Sciences Polytechnic, Huizhou, China; ^2^ College of Nursing, Huizhou Health Sciences Polytechnic, Huizhou, China; ^3^ Institute Office, Huizhou Medicine Institute, Huizhou, China; ^4^ Department of Propaganda and Education, Health Bureau of Huizhou City, Huizhou, China; ^5^ Department of Health, Huizhou First Maternal and Child Health Care Hospital, Huizhou, China; ^6^ Institute Office, Huizhou No. 2 Hospital, Huizhou, China

**Keywords:** depression, insomnia, post-traumatic, cross-sectional study, stress disorder

## Abstract

**Objective:**

This study aimed to assess the depression, anxiety, sleep disorders, and post-traumatic stress disorder (PTSD) among healthcare workers (HCWs) in Huizhou in the post-pandemic period.

**Methods:**

A retrospective multicentric cross-sectional study was conducted from April 25 to May 25, 2023, involving 4,618 HCWs from 46 hospitals in Guangdong Province, China. Psychological well-being was measured using the PHQ-9, GAD-7, Insomnia Severity Index (ISI), and PTSD scales. Chi-square test and multivariable logistic regression were used to identify factors associated with depression, anxiety, insomnia and PTSD.

**Results:**

The rates of anxiety, depression, insomnia, and PTSD were 45.0%, 59.4%, 40.5%, and 10.5%, respectively. HCWs who did not experience negative events were more likely to show anxiety (OR=2.082, 95%CI:1.734-2.499), depression (OR=2.013, 95%CI:1.647-2.460) and insomnia (OR=2.013, 95%CI:1.683-2.409).

**Conclusion:**

There was a high prevalence of anxiety, depression, insomnia, and PTSD in the HCWs after the COVID-19 pandemic.

## 1 Introduction

The COVID-19 outbreak has led to severe physical and mental health issues, with psychological effects often being longer-lasting than physical injuries (1). It places a significant mental burden on frontline healthcare workers (HCWs), leading to depression, anxiety, burnout, and post-traumatic stress disorder (PTSD) among them (2). Indeed, HCWs face serious psychological burdens as their work environment and mental health is complicated and worsened when facing COVID-19 (3). Additionally, systematic reviews and meta-analyses report high rates of anxiety, depression, PTSD, and sleep disorders among HCWs (4, 5).

In December 2022, China faced a nationwide outbreak of Omicron, with increased pressure on HCWs due to policy changes and a rise in infections. HCWs faced high infection rates, relentless workloads, and traumatic conditions, all of which severely impacted their health. As immunity from COVID-19 vaccination and infection wanes over time, infections in China began to rise again in May 2023, fueled by new variants. This surge has placed additional strain on HCWs, exacerbating their workload and psychological stress. However, timely assessments and mental health interventions for frontline HCWs are relatively scarce (6, 7).

Many studies have investigated the mental health and well-being of HCWs in the COVID-19 pandemic (8, 9), however, our understanding of psychological effects in HCWs during the post-COVID-19 pandemic period is still limited due to rarity of studies. The impact of COVID-19 on HCWs varied worldwide due to different political, cultural, environmental, and geographical factors, as well as varying national responses. For instance, over 53% of HCWs in Zimbabwe experienced moderate to severe psychological distress (10), while approximately 40% of frontline nurses in Nigeria suffered from both anxiety and depression in the post-COVID-19 period (11). To our best knowledge, there is a lack of studies exploring anxiety, depression, insomnia, and PTSD among HCWs in Mainland China in the post-COVID-19 period. Therefore, this study was designed to investigate the incidence and risk factors of anxiety, depression, insomnia and PTSD among HCWs during the post-COVID-19 period in Huizhou, China.

## 2 Materials and methods

### 2.1 Participants

This retrospective multicentric cross-sectional study was performed from 25 April 2023 to 25 June 2023 with a random sampling approach in 37 hospitals and health centers community organizations located in 3 districts in Huizhou city, Guangdong Province, China. This survey was conducted during the rising phase of a nationwide COVID-19 outbreak. The respondents completed the electronic questionnaire during the breaks which also reduced unnecessary contact.

The inclusion criteria for participants were as follows: (a) employed HCWs at the hospital, including doctors, nurses, clinical and non-clinical personnels; (b) qualified HCWs possessing a valid Practice Certificate issued by the Ministry of Health of China; (c) voluntary participation in the study; (d) employed for at least 60 days at the end of 2022 during the COVID-19 pandemic. The exclusion criteria were as follows: (a) HCWs with pre-existing mental health conditions, such as schizophrenia, intellectual disabilities, depression, anxiety disorders, or organic diseases affecting the heart, brain, renal functions prior to enrollment; (b) refusal to participate in the survey; (c) incomplete responses in the online questionnaire. The studies involving humans were approved by Research Ethics Committee of Huizhou No. 2 Hospital (No. 2024-025). The studies were conducted in accordance with the local legislation and institutional requirements. The participants provided their written informed consent to participate in this study.

### 2.2 Questionnaire

In the study, data were collected using electronic questionnaires distributed via an online survey APP (https://www.wjx.cn/). The survey was anonymous and took about 10-15 min for each participant. Before distributing the questionnaire form, permission was obtained from department leaders in each hospital, and the researchers explained the purpose and anonymity of the survey to them. After obtaining the informed consent from the hospital staff, department heads sent the questionnaire to eligible HCWs. Two qualified staff reviewed the data. Among the 4,618 distributed questionnaires, 4,528 valid questionnaires (98.85%) were obtained after excluding 90 responses with insufficient data.

### 2.3 Methods

We collected the socio-demographic characteristics of participants, including gender, age, marital status, education, professional title, job type, years of work, hospital degree, health condition, the number of children, having been infected with COVID-19 infection, negative events (infection and sequelae after infection including physical problems; psychological problems; and economic pressure, social isolation, interruption of education, death of relatives and friends), as well as use of psychological support.

Mental health assessments were conducted using the Generalized Anxiety Disorder Scale (GAD-7), the Patient Health Questionnaire (PHQ-9), the Insomnia Severity Index (ISI), as well as the PTSD evaluation.

GAD-7 was adopted to assess the anxiety of each participant based on a four-point Likert scale (0-3) with a total score ranging from 0-21 (12). It is reliable to measure anxiety allied with clinical features showing strong consistency and good test-retest reliability, according to the previous description (13). The scoring criteria were as follows: 0-4 points for no anxiety; 5-9 for mild anxiety; 10-14 points for moderate anxiety, and >15 points for severe anxiety (14). The Cronbach’s alpha of GAD-7 in our study was reliable with a score of 0.947.

Depressive symptoms were assessed based on the PHQ-9 (15), a widely used tool for evaluating depression over the past two weeks. The PHQ-9 consisted of nine items, each scored from 0 (not at all) to 3 (almost every day), with total scores ranging from 0 to 27. Scores are classified as follows: 0-4 for no depression, 5-9 for mild depression, 10-14 for moderate depression, and >15 for severe depression. Its reliability was high in our study with the Cronbach’s alpha value of 0.917.

ISI is used to screen insomnia in general practice, assessing sleep-related issues over the past two weeks, which consists of seven items with a total score of 28 points. A score of 0-7 indicates no insomnia, while scores in a range of 8-14, 15-21, 22-28 suggests subclinical insomnia, moderate insomnia, and severe insomnia, respectively (16). In this study, its reliability was high with a Cronbach’s alpha of 0.941. The cutoff score for insomnia symptoms was set at 8.

The PTSD Checklist for DSM-5-Short Form (PCL-5), a 20-item self-report measure, was utilized to assess the frequency and severity of PTSD symptoms over the past month, using a 5-point Likert scale (0 = not at all; 4 = extremely). Total scores range from 0 to 80, with a cut-off score of 31-33 indicating a provisional PTSD diagnosis (17). In this study, the scale demonstrated strong reliability, with a Cronbach’s alpha of 0.947.

### 2.4 Statistical analysis

Data analysis was conducted using SPSS Statistics (Version 25.0, IBM, USA). Descriptive analysis was used to assess basic demographic data, with frequencies and percentages used to present counting data. Categorical variables were compared using the Chi-square test or Fisher’s exact test. Factors related to the anxiety, depression, ISI, and PTSD were analyzed using a univariate model, followed by multivariable logistic regression. All tests were two-sided, and a P value of <0.05 was considered significant.

## 3 Results

### 3.1 Demographic characteristics

Among the participants, 1,155 (25.51%) were male, with the majority (37.68%) aging in 21-30 years old. The majority were married (69.88%), and in terms of education, most held either an undergraduate degree (47.44%) or a junior college diploma (37.94%). A significant portion (60.14%) had a primary or lower job title. For the occupation, nearly half (44.35%) of the participants were nurses. For the working time per day, over half (52.76%) reported a working duration of more than 10 hr. per day (**Table 1**).

**Table 1:** Characteristics of the enrolled HCWs.

Demographic Variables	Category	n(%)
Gender		
	Male	1155 (25.51)
	Female	3373 (74.49)
Age, years		
	≤ 20	25 (0.55)
	21-30	1706 (37.68)
	31-40	1582 (34.94)
	≥ 41	1215 (26.83)
Marital status		
	Married	3164 (69.88)
	Single	1264 (27.92)
	Divorced	83 (1.83)
	Widow	12 (0.27)
	Separated	5 (0.11)
Education		
	High School/Technical	389 (8.59)
	Junior College	1718 (37.94)
	Undergraduate	2148 (47.44)
	Master and above	273 (6.03)
Professional title		
	Primary	2723 (60.14)
	Intermediate	1288 (28.45)
	Senior	517 (11.42)
Job type		
	Doctor	1439 (31.78)
	Nurse	2008 (44.35)
	Others	1081 (23.87)
Years of work		
	< 1 year	184 (4.06)
	1-5 years	1042 (23.01)
	6-10 years	913 (20.16)
	> 10 years	2389 (52.76)
Hospital type		
	Tertiary	2131 (47.06)
	Secondary	795 (17.56)
	Primary	1602 (35.38)
Health condition		
	Excellent	1348 (29.77)
	Good	2269 (50.11)
	Fair	847 (18.71)
	Poor	64 (1.41)
Single-child family		
	Yes	451 (9.96)
	No	4077 (90.04)
Number of children		
	None	1544 (34.1)
	1	1322 (29.2)
	≥2	1662 (36.7)
Infected with COVID-19		
	Yes	3925 (86.68)
	No	331 (7.31)
	Unknown	272 (6.01)
Negative events		
	Yes	667 (14.73)
	No	3861 (85.27)
Use of psychological support		
	Yes	507 (11.2)
	No	4021 (88.8)

### 3.2 Univariate logistic regression analysis

Among the 4,528 participants, the prevalence of anxiety, depression, insomnia, and PTSD symptoms was 45.0%, 59.4%, 40.5%, and 10.5%, respectively. For the univariate logistic regression analysis of anxiety symptoms, the variables with a P value of less than 0.05 were adopted including gender, age, education, professional title, job type, years of work, hospital type, health condition, number of children, negative events, as well as use of psychological support during COVID-19. For the depression symptoms, the adopted variables with a P value of less than 0.05 were gender, age, education, job type, hospital type, years of work, health condition, number of children, negative events, as well as use of psychological support during COVID-19. For insomnia, the variables with a P value of less than 0.05 included education, professional title, job type, years of work, hospital type, health condition, negative events, as well as use of psychological support during COVID-19. The variables for the PTSD symptoms with a P value of less than 0.05 included age, education, job type, years of work, health condition, negative events, and use of psychological support during COVID-19 (**Supplementary Tables 1**-**4**).

### 3.3 Multivariate logistic regression analysis

These statistically significant variables in univariate analysis were then adopted in the multivariate logistic regression analysis. **Table 2** presented a multivariable logistic regression analysis of factors related to anxiety symptoms among participants. HCWs aged over 41 years old were more likely to experience anxiety than those under 20 (OR=1.435, 95%CI: 1.182-1.742). Compared to those with a high school or technical school education, those with a junior college education were less likely to report anxiety (OR=0.570, 95%CI: 0.385-0.842). Regarding professional titles, HCWs with intermediate titles were less likely to report anxiety compared to those with primary or lower titles (OR=0.722, 95%CI: 0.556-0.093). HCWs with 1-5 years of work experience were also less likely to report anxiety than those with less than 1 year of experience (OR=0.557, 95%CI: 0.364-0.852). Those in good, fair, or poor health, compared to excellent health, were more likely to experience anxiety symptoms. HCWs with two or more children were also more likely to report anxiety than those with no children. Additionally, HCWs who had not experienced negative events (OR=2.082, 95%CI: 1.734-2.499) were more likely to show anxiety. Unexpectedly, HCWs with no psychological support were likely to show reduced risk of anxiety (OR=0.889, 95%CI: 0.727-1.087).

**Table 2:** Multivariable logistic regression analysis of related factors of anxiety symptoms among the HCWs.

Variables	OR	P value	95%CI
Gender			
Male	–	–	–
Female	0.848	0.060	0.715-1.007
Age, years			
≤20	–	–	–
21-30	1.173	0.741	0.045-3.030
31-40	1.261	0.128	0.936-1.698
≥41	1.435	<0.001	1.182-1.742
Education			
High School or Technical School	–	–	–
Junior college	0.570	0.005	0.385-0.842
Undergraduate	0.765	0.098	0.557-1.050
Master and above	0.835	0.229	0.623-1.120
Professional Title			
Primary and below	–	–	–
Intermediate	0.722	0.014	0.556-0.093
Senior	0.800	0.070	0.629-1.018
Job type			
Doctor	–	–	–
Nurse	1.357	0.137	0.907-2.029
Others	1.505	0.759	1.017-2.227
Years of work			
Less than 1	–	–	–
1-5	0.557	0.007	0.364-0.852
6-10	0.999	0.995	0.757-1.319
More than 10	1.009	0.940	0.806-1.263
Hospital degree			
Tertiary	–	–	–
Secondary	1.024	0.764	0.877-1.196
Primary	1.059	0.544	0.880-1.274
Health condition			
Excellent	–	–	–
Good	0.083	<0.001	0.040-0.171
Fair	0.165	<0.001	0.080-0.341
Poor	0.379	0.009	0.182-0.789
Number of children			
None	–	–	–
1	1.039	0.723	0.843-1.280
≥2	0.833	0.026	0.709-0.979
Negative events			
Yes	–	–	–
No	2.082	<0.001	1.734-2.499
Use of psychological support			
Yes	–	–	–
No	0.889	0.025	0.727-1.087

**Table 3** showed the multivariable logistic regression analysis of related factors of depression among the participants. Compared with male HCWs, female HCWs showed reduced risks of depression (OR=0.744, 95%CI: 0.627-0.883). HCWs of junior college showed reduced risks of developing depression compared with the high school or technical school HCWs (OR=0.561, 95%CI:0.382-0.823). In addition, HCWs who did not experience negative events showed increased risks of depression (OR=2.013, 95%CI:1.647-2.460) and those underwent no psychological support showed reduced risks of depression (OR=0.798, 95%CI:0.654-0.973).

**Table 3:** Multivariable logistic regression analysis of related factors of depressive symptoms among the HCWs.

Variables	OR	P value	95%CI
Gender			
Male	–	–	–
Female	0.744	0.001	0.627-0.883
Age, years			
≤20	–	–	–
21-30	1.152	0.757	0.469-2.832
31-40	1.214	0.194	0.906-1.625
≥41	1.284	0.008	1.068-1.543
Education			
High School or Technical School	–	–	–
Junior college	0.561	0.003	0.382-0.823
Undergraduate	0.737	0.061	0.535-1.014
Master and above	0.931	0.643	0.689-1.258
Job type			
Doctor	–	–	–
Nurse	1.347	0.123	0.922-1.968
Others	1.341	0.119	0.928-1.939
Years of work			
Less than 1	–	–	–
1-5	0.567	0.007	0.376-0.854
6-10	1.073	0.624	0.810-1.421
More than 10	1.940	0.595	0.748-1.181
Hospital degree			
Tertiary	–	–	–
Secondary	1.061	0.451	0.909-1.238
Primary	1.149	0.147	0.953-1.385
Health condition			
Excellent	–	–	–
Good	0.110	<0.001	0.049-0.245
Fair	0.248	0.001	0.111-0.554
Poor	0.617	0.245	0.273-1.394
Number of children			
None	–	–	–
1	1.151	0.199	0.929-1.428
≥2	0.887	0.147	0.755-1.043
Negative events			
Yes	–	–	–
No	2.013	<0.001	1.647-2.460
Use of psychological support			
Yes	–	–	–
No	0.798	0.026	0.654-0.973

The others in the job type included pre-examination and triage, community health monitoring, vaccination, emergency preparation, medical supplies management, psychological assistance, and scientific researchers.

In terms of insomnia, compared with years of work less than 1, HCWs worked for 1-5 years were less likely to show insomnia (OR=0.536, 95%CI:0.369-0.777). Compared to HCWs working in the tertiary hospitals, those worked in the secondary hospitals showed increased risks of insomnia (OR=1.236, 95%CI:1.056-1.446). HCWs who did not experience negative events were more likely to show insomnia (OR=2.013, 95%CI:1.683-2.409, **Table 4**).

**Table 4:** Multivariable logistic regression analysis of related factors of insomnia symptoms among the HCWs.

Variables	OR	P value	95% CI
Education Level			
High School or Technical School	–	–	–
Junior College	0.635	0.021	0.432-0.934
Undergraduate	0.811	0.192	0.592-1.111
Master and above	0.846	0.262	0.631-1.134
Professional title			
Primary and below	–	–	–
Intermediate	0.961	0.755	0.750-1.232
Senior	1.060	0.618	0.843-1.334
Job type			
Doctor	–	–	–
Nurse	1.341	0.153	0.896-2.006
Others	1.423	0.074	0.966-2.095
Years of work			
Less than 1	–	–	–
1-5	0.536	0.001	0.369-0.777
6-10	0.970	0.747	0.803-1.170
More than 10	0.861	0.107	0.717-1.033
Hospital degree			
Tertiary	–	–	–
Secondary	1.236	0.008	1.056-1.446
Primary	1.199	0.058	0.994-1.447
Health condition			
Excellent	–	–	–
Good	0.057	<0.001	0.027-0.121
Fair	0.122	<0.001	0.058-0.260
Poor	0.290	0.001	0.135-0.621
Negative events			
Yes	–	–	–
No	2.013	<0.001	1.683-2.409
Use of psychological support			
Yes	–	–	–
No	0.900	0.313	0.734-1.104

HCWs over 20 years old had a higher risk of PTSD symptoms compared to those 20 or younger. Similarly, those with good, fair, or poor health were more likely to report PTSD symptoms compared to those in excellent health. Lastly, HCWs experienced no negative events were also more likely to show PTSD symptoms (OR=2.383, 95% CI: 1.889-3.006), while those with no psychological support showed reduced risk of PTSD symptoms (OR=0.646, 95% CI: 0.436-0.956, **Table 5**).

**Table 5:** Multivariable logistic regression analysis of related factors of PTSD among the HCWs.

Variables	OR	P value	95% CI
Age, years			
≤20	–	–	–
21-30	8.467	<0.001	2.602-27.559
31-40	1.699	0.022	1.078-2.677
≥ 41	1.930	<0.001	1.416-2.631
Education level			
High school or technical school	–	–	–
Junior college	0.867	0.652	0.466-1.614
Undergraduate	1.055	0.824	0.657-1.693
Master and above	1.069	0.774	0.679-1.681
Job Type			
Doctor	–	–	–
Nurse	2.906	0.029	1.112-7.592
Others	1.390	0.780	0.137-14.056
Years of work			
Less than 1	–	–	–
1-5	0.382	0.021	0.169-0.862
6-10	0.926	0.714	0.615-1.396
More than 10	1.101	0.569	0.791-1.531
Health condition			
Excellent	–	–	–
Good	0.071	<0.001	0.040-0.127
Fair	0.111	<0.001	0.065-0.191
Poor	0.396	0.001	0.229-0.674
Negative events			
Yes	–	–	–
No	2.383	<0.001	1.889-3.006
Use of psychological support			
Yes	–	–	–
No	0.646	0.029	0.436-0.956

## 4 Discussion

This study examined the effects of the COVID-19 pandemic on frontline HCWs in Huizhou after the COVID-19 prevention and control policies in China. We assessed anxiety, depression, PTSD, and sleep quality of the HCWs, which revealed that the prevalence rates of anxiety, depression, insomnia, and PTSD were 45.0%, 59.4%, 40.5% and 10.5%, respectively. These rates were lower than those observed among Chinese HCWs during the peak of the pandemic and also lower than Xiao’s study, which reported 62.51% for anxiety, 69.20% for depression, and 76.78% for insomnia among nurses (18). One possible explanation was that HCWs in our study were more prepared during this period to reduce their anxiety, depression, insomnia, and PTSD. Over time, their fear and anxiety diminished as they adapted to the ongoing nature of COVID-19. However, mental health issues can persist long after recovery (19). Therefore, continued evaluation of the mental health of COVID-19 survivors is essential.

In the COVID-19 crisis, frontline HCWs encountered immense physical and mental pressure. They managed a growing number of patients, faced risks of infection, and struggled with limited resources and emotional support (20). In addition, COVID-19 infection would further increase their anxiety and depression. Similarly, the general public is also at risk of psychological distress, increasing the overall anxiety burden among the HCWs. The sudden shortage of HCWs and the number surge in cases, especially after the full liberation of COVID-19 prevention, have increased pressure on HCWs to provide adequate care in Mainland China. In the presence of high COVID-19 case numbers per capita, junior doctors in the US who directly cared for patients with a COVID-19 diagnosis experienced elevated levels of anxiety and depression (21). In contrast, in a study performed in Australia, the authors indicated that junior doctors were mentally well and able to function at a high level during a global pandemic (22). In our study, multivariable logistic regression analysis revealed that junior HCWs, particularly those with 1-5 years of experience or 2 or more children, showed reduced risks of developing anxiety and depression compared with the other counterparts. Female HCWs were more prone to show anxiety and depression than their male counterparts, possibly due to greater sensitivity, weaker psychological endurance, and the burden of balancing work with childcare and household duties (23). Consistently, they reported higher rates of depression during COVID-19, influenced by individual factors like lack of social support and organizational factors such as heavy workloads and lack of recognition. However, some studies highlight specific situations where male HCWs experienced higher levels of psychological distress. For example, unemployment and isolation due to quarantine had a more pronounced negative effect on mental health in male HCWs, particularly increasing depression and anxiety levels. The role of employment appears crucial in influencing male emotional states, with unemployed male HCWs more severely impacted by the psychological effects (24, 25). In this study, female HCWs did not show increased risks of anxiety and depression compared with the male counterparts. In the future, more sample sized studies are required to further illustrate the sex differences in depression and anxiety during the post-COVID-19 period.

Various psychosocial factors during COVID-19, such as stress, anxiety, depression, workload, and uncertainty about disease control, significantly impacted sleep quality. In a previous study, grassroots HCWs, facing increased responsibilities after community management phase, experienced greater work pressure and sleep deprivation (26). Additionally, reduced income during the pandemic contributed to insomnia, especially among those with low annual income and 1-5 years of experience (27). Our data showed that HCWs in secondary hospitals were more likely to show insomnia (OR=1.236, 95%CI:1.056-1.446), as their incomes were lower compared with those in the tertiary hospitals. In some cases, the prevalence of insomnia among frontline HCWs reached 30% to 60% during the peak of the crisis (28). This significant rise in sleep disturbances was driven by increased workload, prolonged exposure to high-stress environments, fear of infection, and emotional fatigue from witnessing severe patient outcomes and death. These challenges have underscored the need for targeted interventions to address the sleep-related and broader psychological health challenges faced by HCWs during and after the pandemic (29, 30). In a previous study focused on the combined impact of gender and age on PTSD and insomnia during COVID-19 outbreak in China, Liu et al. reported that females above 50 years of age showed a lower level of depressive symptoms (OR = 0.448, 95%CI: 0.220–0.911) compared with females aged 18–25 (31). Consistently, our data showed that HCWs aged 21 or more showed increased risks of PTSD compared with those aged less than 20 years. Existing literatures showed that poor health generally increased the risk of PTSD. For instance, HCWs who experienced physical exhaustion, illness, or long-term health complications were often more susceptible to PTSD because they had fewer physical and psychological resources to cope with the trauma (32). Unexpectedly, our data showed that HCWs with good, fair or poor conditions showed reduced risks of developing PTSD compared with these with excellent health conditions. One possible explanation was that those with excellent conditions were more likely to take more tasks during and after COVID-19, which may face more challenges than others. In the future, we will expand our sample size to illustrate this. HCWs who did not experience negative events (e.g. physical and mental problems, financial pressure, death of relatives and friends) were more likely to show anxiety (OR=2.082, 95%CI:1.734-2.499), depression (OR=2.013, 95%CI:1.647-2.460) and insomnia (OR=2.013, 95%CI:1.683-2.409). This may reveal the complexity of mental health mechanisms under the special context of the epidemic. This phenomenon needs to be explained in multiple dimensions. For example, long-term exposure to high-risk environments, uncertainty about the direction of the epidemic, and other persistent low-intensity stress are not included in negative events, but cause psychological symptoms through cumulative effects. Although they have not experienced negative events, excessive alertness to potential risks leads to continuous activation of the sympathetic nervous system, resulting in psychological reactions similar to anxiety, depression and insomnia. It is also possible that those who have experienced negative events can gain growth resilience by reconstructing cognition, while those who have not experienced it lack this opportunity for psychological reconstruction and instead fall into a sense of meaninglessness. In short, in extreme stress situations, we need to pay attention to the identification of implicit stress.

Several limitations of our study should be noted. First, as it is based on cross-sectional data, no causal relationships can be inferred, and ongoing changes in psychological status of HCWs were not examined. Longer-term research may yield more accurate results. Second, the use of self-reported scales is limited by perceptions and potential biases of each participant in survey distribution. Third, self-reported psychological responses may be affected by recall bias, highlighting the need for more qualitative and in-depth studies in this area. Fourth, this is a retrospective analysis, and we could not analyze the long-term effects in the participants.

## 5 Conclusion

In this study, we investigated the prevalence of anxiety, depression, insomnia, and PTSD in the HCWs after the COVID-19 pandemic. Our study reported prevalence rates of 45.0% for anxiety, 59.4% for depression, 40.5% for insomnia, and 10.5% for PTSD. In addition, we also screened the risk factors for anxiety, depression, insomnia and PTSD.

## Data Availability

The original contributions presented in the study are included in the article/**Supplementary Material**. Further inquiries can be directed to the corresponding author/s.
