# Supplementary material for: Psychological status of healthcare workers in the post-COVID 19 period in China: a retrospective multicentric cross-sectional study
Source: Front Psychiatry. 2025 Apr 7;16:1520361. doi: 10.3389/fpsyt.2025.1520361 (PMC12009875; doi:10.3389/fpsyt.2025.1520361)
Supplement: Supplementary file 1 [file Table1.docx]

**Supplementary Table 1 Univariates analysis of anxiety symptoms of the HCWs**

| Demographic Variables | Anxiety symptoms <5 (n = 2489) | Anxiety symptoms ≥5 (n = 2039) | χ^2^ | P value |
| --- | --- | --- | --- | --- |
| Gender |  |  | 12.749 | <0.001 |
| Male | 687 (59.5%) | 468 (40.5%) |  |  |
| Female | 1802 (53.4) | 1571 (46.6%) |  |  |
| Age (years) |  |  | 37.052 | <0.001 |
| ≤20 | 17 (68.0%) | 8 (32.0%) |  |  |
| 21-30 | 933 (54.7%) | 773 (45.3%) |  |  |
| 31-40 | 793 (50.1%) | 789 (49.9%) |  |  |
| ≥ 41 | 746 (61.4%) | 469 (38.6%) |  |  |
| Marital status |  |  |  |  |
| Married | 1747 (55.2%) | 1417 (44.8%) | 4.599 | 0.331 |
| Single | 682 (54.0%) | 582 (46.0%) |  |  |
| Divorced | 53 (63.9%) | 30 (36.1%) |  |  |
| Widow | 5 (41.7%) | 7 (58.3%) |  |  |
| Separated | 2 (40.0%) | 3 (60.0%) |  |  |
| Education |  |  | 42.220 | <0.001 |
| High School or Technical School | 262 (67.4%) | 127 (32.6%) |  |  |
| Junior college | 985 (57.3%) | 733 (42.7%) |  |  |
| Undergraduate | 1110 (51.7%) | 1038 (48.3%) |  |  |
| Master and above | 132 (48.4%) | 141 (51.6%) |  |  |
| Professional title | |  | 8.290 | 0.016 |
| Primary and below | 1544 (56.7%) | 1179 (43.3%) |  |  |
| Intermediate | 674 (52.3%) | 614 (47.7%) |  |  |
| Senior | 271 (52.4%) | 246 (47.6%) |  |  |
| Job type |  |  | 17.345 | <0.001 |
| Doctor | 803 (55.8%) | 636 (44.2%) |  |  |
| Nurse | 1042 (51.9%) | 966 (48.1%) |  |  |
| Others | 644 (59.6%) | 437 (40.4%) |  |  |
| Years of work |  |  | 19.736 | <0.001 |
| Less than 1 | 127 (69.0%) | 57 (31.0%) |  |  |
| 1-5 | 560 (53.7%) | 482 (46.3%) |  |  |
| 6-10 | 472 (51.7%) | 441 (48.3%) |  |  |
| More than 10 | 1330 (55.7%) | 1059 (44.3%) |  |  |
| Hospital Degree |  |  | 10.007 | 0.007 |
| Tertiary | 1125 (52.8%) | 1006 (47.2%) |  |  |
| Secondary | 435 (54.7%) | 360 (45.3%) |  |  |
| Primary | 929 (58.0%) | 673 (42.0%) |  |  |
| Health Condition |  |  | 350.013 | <0.001 |
| Excellent | 964 (71.5%) | 384 (28.5%) |  |  |
| Good | 1232 (54.3%) | 1037 (45.7%) |  |  |
| Fair | 284 (33.5%) | 563 (66.5%) |  |  |
| Poor | 9 (14.1%) | 55 (85.9%) |  |  |
| One-child Families |  |  | 0.043 | 0.835 |
| Yes | 250 (55.4%) | 201 (44.6%) |  |  |
| No | 2239 (54.9%) | 1838 (45.1%) |  |  |
| Number of Children |  |  | 8.487 | 0.014 |
| No children | 829 (53.7%) | 715 (46.3%) |  |  |
| 1 | 771 (58.3%) | 551 (41.7%) |  |  |
| More than 2 | 889 (53.5%) | 773 (46.5%) |  |  |
| Infected with COVID-19 |  |  |  |  |
| Yes | 2138 (54.5%) | 1787 (45.5%) | 2.962 | 0.227 |
| No | 192 (58.0%) | 139 (42.0%) |  |  |
| Not sure | 159 (58.5%) | 113 (41.5%) |  |  |
| Negative Events |  |  | 113.925 | <0.001 |
| Yes | 240 (36.0%) | 427 (64.0%) |  |  |
| No | 2249 (58.2%) | 1612 (41.8%) |  |  |
| Use of Psychological Support |  |  |  |  |
| Yes | 307 (60.6%) | 200 (39.4%) | 7.190 | 0.007 |
| No | 2182 (54.3%) | 1839 (45.7%) |  |  |

**Supplementary Table 2 Univariates analysis of depression of the HCWs**

| Demographic variables | Depression symptoms<5 (n = 1837) | Depression symptoms≥5 (n =2691) | χ^2^ | P value |
| --- | --- | --- | --- | --- |
| Gender |  |  | 28.151 | <0.001 |
| Male | 545 (47.2%) | 610 (52.8%) |  |  |
| Female | 1292 (38.3%) | 2081 (61.7%) |  |  |
| Age(y) |  |  | 44.348 | <0.001 |
| ≤20 | 13 (52.0%) | 12 (48.0%) |  |  |
| 21-30 | 644 (37.7%) | 1062 (62.3%) |  |  |
| 31-40 | 592 (37.4%) | 990 (62.6%) |  |  |
| ≥ 41 | 588 (48.4%) | 627 (51.6%) |  |  |
| Marital status |  |  | 8.902 | 0.064 |
| Married | 1318 (41.7%) | 1846 (58.3%) |  |  |
| Single | 475 (37.6%) | 789 (62.4%) |  |  |
| Divorced | 39 (47.0%) | 44 (53.0%) |  |  |
| Widow | 4 (33.3%) | 8 (66.7%) |  |  |
| Separated | 1 (20.0%) | 4 (80.0%) |  |  |
| Education |  |  | 45.493 | <0.001 |
| High School or Technical School | 207 (53.2%) | 182 (46.8%) |  |  |
| Junior college | 741 (43.1%) | 977 (56.9%) |  |  |
| Undergraduate | 790 (36.8%) | 1358 (63.2%) |  |  |
| Master and above | 99 (36.3%) | 174 (63.7%) |  |  |
| Professional Title |  |  | 3.179 | 0.204 |
| Primary and below | 1128 (41.4%) | 1595 (58.6%) |  |  |
| Intermediate | 496 (38.5%) | 792 (61.5%) |  |  |
| Senior | 213 (41.2%) | 304 (58.8%) |  |  |
| Job type |  |  | 14.146 | 0.001 |
| Doctor | 606 (42.1%) | 833 (57.9%) |  |  |
| Nurse | 755 (37.6%) | 1253 (62.4%) |  |  |
| Others | 476 (44.0%) | 605 (56.0%) |  |  |
| Years of work |  |  | 29.150 | <0.001 |
| Less than 1 | 97 (52.7%) | 87 (47.3%) |  |  |
| 1-5 | 374 (35.9%) | 668 (64.1%) |  |  |
| 6-10 | 343 (37.6%) | 570 (62.4%) |  |  |
| More than 10 | 1023 (42.8%) | 1366 (57.2%) |  |  |
| Hospital degree |  |  | 11.714 | 0.003 |
| Tertiary | 825 (38.7%) | 1306 (61.3%) |  |  |
| Secondary | 308 (38.7%) | 487 (61.3%) |  |  |
| Primary | 704 (43.9%) | 898 (56.1%) |  |  |
| Health condition |  |  | 389.183 | <0.001 |
| Excellent | 803 (59.6%) | 545 (40.4%) |  |  |
| Good | 863 (38.0%) | 1406 (62.0%) |  |  |
| Fair | 164 (19.4%) | 683 (80.6%) |  |  |
| Poor | 7 (10.9%) | 57 (89.1%) |  |  |
| One-child families |  |  | 1.993 | 0.158 |
| Yes | 169 (37.5%) | 282 (62.5%) | 0.158 |  |
| No | 1668 (40.9%) | 2409 (59.1%) |  |  |
| Number of children |  |  | 16.337 | <0.001 |
| No children | 568 (36.8%) | 976 (63.2%) |  |  |
| 1 | 583 (44.1%) | 739 (55.9%) |  |  |
| More than 2 | 686 (41.3%) | 976 (58.7%) |  |  |
| Having been infected with COVID-19 |  |  | 4.321 | 0.115 |
| Yes | 1570 (40.0%) | 2355 (60.0%) |  |  |
| No | 143 (43.2%) | 188 (56.8%) |  |  |
| Not sure | 124 (45.6%) | 148 (54.4%) |  |  |
| Negative events |  |  | 81.321 | <0.001 |
| Yes | 165 (24.7%) | 502 (75.3%) |  |  |
| No | 1672 (43.3%) | 2189 (56.7%) |  |  |
| Use of psychological support during COVID-19 |  |  | 15.721 | <0.001 |
| Yes | 247 (48.7%) | 260 (51.3%) |  |  |
| No | 1590 (39.5%) | 2431 (60.5%) |  |  |

**Supplementary Table 3 Univariates analysis of insomnia of the HCWs**

| Demographic variables | Insomnia symptoms <8 (n = 2692) | Insomnia symptoms ≥8 (n =1836) | χ^2^ | P value |
| --- | --- | --- | --- | --- |
| Gender |  |  | 0.334 | 0.563 |
| Male | 695 (60.2%) | 460 (39.8%) |  |  |
| Female | 1997 (59.2%) | 1376 (40.8%) |  |  |
| Age(y) |  |  | 6.652 | 0.084 |
| ≤20 | 17 (68.0%) | 8 (32.0%) |  |  |
| 21-30 | 1031 (60.4%) | 675 (39.6%) |  |  |
| 31-40 | 902 (57.0%) | 680 (43.0%) |  |  |
| ≥41 | 742 (61.1%) | 473 (38.9%) |  |  |
| Marital status |  |  | 2.111 | 0.715 |
| Married | 1888 (59.7%) | 1276 (40.3%) |  |  |
| Single | 742 (58.7%) | 522 (41.3%) |  |  |
| Divorced | 52 (62.7%) | 31 (37.3%) |  |  |
| Widow | 6 (50.0%) | 6 (50.0%) |  |  |
| Separated | 4 (80.0%) | 1 (20.0%) |  |  |
| Education |  |  | 22.488 | <0.001 |
| High School or Technical School | 263 (67.6%) | 126 (32.4%) |  |  |
| Junior college | 1054 (61.4%) | 664 (38.6%) |  |  |
| Undergraduate | 1231 (57.3%) | 917 (42.7%) |  |  |
| Master and above | 144 (52.7%) | 129 (47.3%) |  |  |
| Professional Title |  |  | 11.301 | 0.004 |
| Primary and below | 1673 (61.4%) | 1050 (38.6%) |  |  |
| Intermediate | 724 (56.2%) | 564 (43.8%) |  |  |
| Senior | 295 (57.1%) | 222 (42.9%) |  |  |
| Job type |  |  | 15.606 | <0.001 |
| Doctor | 845 (58.7%) | 594 (41.3%) |  |  |
| Nurse | 1150 (57.3%) | 858 (42.7%) |  |  |
| Others | 697 (64.5%) | 384 (35.5%) |  |  |
| Years of work |  |  | 15.592 | 0.001 |
| Less than 1 | 135 (73.4%) | 49 (26.6%) |  |  |
| 1-5 | 611 (58.6%) | 431 (41.4%) |  |  |
| 6-10 | 543 (59.5%) | 370 (40.5%) |  |  |
| More than 10 | 1403 (58.7%) | 986 (41.3%) |  |  |
| Hospital degree |  |  | 15.541 | <0.001 |
| Tertiary | 1212 (56.9%) | 919 (43.1%) |  |  |
| Secondary | 467 (58.7%) | 328 (41.3%) |  |  |
| Primary | 1013 (63.2%) | 589 (36.8%) |  |  |
| Health condition |  |  | 392.088 | <0.001 |
| Excellent | 1025 (76.0%) | 323 (24.0%) |  |  |
| Good | 1347 (59.4%) | 922 (40.6%) |  |  |
| Fair | 312 (36.8%) | 535 (63.2%) |  |  |
| Poor | 8 (12.5%) | 56 (87.5%) |  |  |
| One-child families |  |  | 0.008 | 0.930 |
| Yes | 269 (59.6%) | 182 (40.4%) |  |  |
| No | 2423 (59.4%) | 1654 (40.6%) |  |  |
| Number of children |  |  | 1.445 | 0.485 |
| No children | 910 (58.9%) | 634 (41.1%) |  |  |
| 1 | 804 (60.8%) | 518 (39.2%) |  |  |
| More than 2 | 978 (58.8%) | 684 (41.2%) |  |  |
| Having been infected with COVID-19 | |  | 0.194 | 0.908 |
| Yes | 2337 (59.5%) | 1588 (40.5%) |  |  |
| No | 193 (58.3%) | 138 (41.7%) |  |  |
| Not sure | 162 (59.6%) | 110 (40.4%) |  |  |
| Negative events |  |  | 116.801 | <0.001 |
| Yes | 270 (40.5%) | 397 (59.5%) |  |  |
| No | 2422 (62.7%) | 1439 (37.3%) |  |  |
| Use of psychological support during COVID-19 | |  | 5.565 | 0.018 |
| Yes | 326 (64.3%) | 181 (35.7%) |  |  |
| No | 2366 (58.8%) | 1655 (41.2%) |  |  |

**Supplementary Table 4 Univariates analysis of PTSD of the HCWs**

| Demographic variables | PTSD symptoms <32 (n = 4054) | PTSD symptoms ≥33 (n =474 ) | χ^2^ | P value |
| --- | --- | --- | --- | --- |
| Gender |  |  | 0.054 | 0.816 |
| Male | 1032 (89.4%) | 123 (10.6%) |  |  |
| Female | 3022 (89.6%) | 351 (10.4%) |  |  |
| Age(y) |  |  | 31.261 | <0.001 |
| ≤20 | 20 (80.0%) | 5 (20.0%) |  |  |
| 21-30 | 1522 (89.2%) | 184 (10.8%) |  |  |
| 31-40 | 1379 (87.2%) | 203 (12.8%) |  |  |
| ≥41 | 1133 (93.3%) | 82 (6.7%) |  |  |
| Marital status |  |  | 5.987 | 0.200 |
| Married | 2855 (90.2%) | 309 (9.8%) |  |  |
| Single | 1111 (87.9%) | 153 (12.1%) |  |  |
| Divorced | 74 (89.2%) | 9 (10.8%) |  |  |
| Widow | 10 (83.3%) | 2 (16.7%) |  |  |
| Separated | 4 (80.0%) | 1 (20.0%) |  |  |
| Education level |  |  | 9.940 | 0.019 |
| High School or Technical School | 363 (93.3%) | 26 (6.7%) |  |  |
| Junior college | 1550 (90.2%) | 168 (9.8%) |  |  |
| Undergraduate | 1899 (88.4%) | 249 (11.6%) |  |  |
| Master and above | 242 (88.6%) | 31 (11.4%) |  |  |
| Professional Title |  |  |  |  |
| Primary and below | 2433 (89.3%) | 290 (10.7%) | 1.957 | 0.376 |
| Intermediate | 1149 (89.2%) | 139 (10.8%) |  |  |
| Senior | 472 (91.3%) | 45 (8.7%) |  |  |
| Job type |  |  | 14.640 | 0.001 |
| Doctor | 1280 (89.0%) | 159 (11.0%) |  |  |
| Nurse | 1773 (88.3%) | 235 (11.7%) |  |  |
| Others | 1001 (92.6%) | 80 (7.4%) |  |  |
| Years of work |  |  | 15.710 | 0.001 |
| Less than 1 | 175 (95.1%) | 9 (4.9%) |  |  |
| 1-5 | 928 (89.1%) | 114 (10.9%) |  |  |
| 6-10 | 792 (86.7%) | 121 (13.3%) |  |  |
| More than 10 | 2159 (90.4%) | 230 (9.6%) |  |  |
| Hospital degree |  |  | 2.166 | 0.339 |
| Tertiary | 1893 (88.9%) | 238 (11.2%) |  |  |
| Secondary | 715 (89.9%) | 80 (10.1%) |  |  |
| Primary | 1446 (90.3%) | 156 (9.7%) |  |  |
| Health condition |  |  | 332.104 | <0.001 |
| Excellent | 1285 (95.3%) | 63 (4.7%) |  |  |
| Good | 2093 (92.2%) | 176 (7.8%) |  |  |
| Fair | 643 (75.9%) | 204 (24.1%) |  |  |
| Poor | 33 (51.6%) | 31 (48.4%) |  |  |
| One-child families |  |  | 0.602 | 0.438 |
| Yes | 399 (88.5%) | 52 (11.5%) |  |  |
| No | 3655 (89.6%) | 422 (10.4%) |  |  |
| Number of children |  |  | 5.012 | 0.082 |
| No children | 1361 (88.1%) | 183 (11.9%) |  |  |
| 1 | 1197 (90.5%) | 125 (9.5%) |  |  |
| More than 2 | 1496 (90.0%) | 166 (10.0%) |  |  |
| Having been infected with COVID-19 | |  | 1.450 | 0.484 |
| Yes | 3519 (89.7%) | 406 (10.3%) |  |  |
| No | 290 (87.6%) | 41 (12.4%) |  |  |
| Not sure | 245 (90.1%) | 27 (9.9%) |  |  |
| Negative events |  |  | 126.687 | <0.001 |
| Yes | 515 (77.2%) | 152 (22.8%) |  |  |
| No | 3539 (91.7%) | 322 (8.3%) |  |  |
| Use of psychological support during COVID-19 | |  | 11.547 | <0.001 |
| Yes | 476 (93.9%) | 31 (6.1%) |  |  |
| No | 3578 (89.0%) | 443 (11.0%) |  |  |
